# H60: A Unique Murine Hematopoietic Cell-Restricted Minor Histocompatibility Antigen for Graft-versus-Leukemia Effect

**DOI:** 10.3389/fimmu.2020.01163

**Published:** 2020-06-10

**Authors:** Eun Young Choi, Kyungho Choi, Giri Nam, Woojin Kim, Minho Chung

**Affiliations:** ^1^Department of Biomedical Sciences, Seoul National University College of Medicine, Seoul, South Korea; ^2^Institute of Human Environment Interface Biology, Seoul National University College of Medicine, Seoul, South Korea; ^3^Department of Biochemistry and Molecular Biology, Seoul National University College of Medicine, Seoul, South Korea; ^4^Cancer Research Institute, Seoul National University College of Medicine, Seoul, South Korea

**Keywords:** H60, minor histocompatibility antigen, graft-versus-leukemia, graft-versus-host-disease, hematopoieic cell-restricted antigen

## Abstract

Allogeneic hematopoietic stem cell transplantation (allo-HSCT) is an important treatment for many types of hematological malignancies. Matching of donor and recipient for the major histocompatibility complex (MHC) improves the HSCT reconstitution, but donor-derived T cells reactive to non-MHC encoded minor histocompatibility antigens (MiHAs) can induce graft-versus-host disease (GVHD) while also being needed for graft-versus-leukemia (GVL) effects. MiHAs are allelically variant self-peptides presented conventionally on MHC molecules, but are alloantigenic in transplantation settings. Immunodominant MiHAs are most strongly associated with GVHD and GVL. There is need for mouse paradigms to understand these contradictory effects. H60 is a highly immunodominant mouse MiHA with hematopoietic cell-restricted expression. Immunodominance of H60 is tightly associated with its allelic nature (presence vs. absence of the transcripts), and the qualitative (TCR diversity) and quantitative (frequency) traits of the reactive T cells. The identity as a hematopoietic cell-restricted antigen (HRA) of H60 assists the appearance of the immunodominace in allo-HSCT circumstances, and generation of GVL effects without induction of serious GVHD after adoptive T cell transfer. Also it allows the low avidity T cells to escape thymic negative selection and exert GVL effect in the periphery, which is a previously unevaluated finding related to HRAs. In this review, we describe the molecular features and immunobiology in detail through which H60 selectively exerts its potent GVL effect. We further describe how lessons learned can be extrapolated to human allo-HCST.

## Introduction

Allogeneic hematopoietic stem cell transplantation (allo-HSCT) was originally developed as a means to reconstitute the immune system of patients with hematological malignancies after anti-tumor radio/chemotherapy (1). T cell repopulation shortly after transplantation is attributed to the expansion of mature T cells from the donor bone marrow (BM) inoculum, rather than *de novo* T cell regeneration (2). Ideally, these mature donor-derived T cells confer rapid protection from infection following allo-HSCT, while also being cytotoxic to residual tumor cells. This latter phenomenon is referred to as the graft-versus-leukemia (GVL) effect (3). Thus, allo-HSCT is considered as an anti-tumor treatment modality beyond its immune reconstitution capability. Mechanistically, donor-derived mature T cells elicit the GVL effect via recognition of host allo-antigens expressed by hematopoietic tumor cells (4). The downside is that they can also attack normal host tissues expressing allo-antigens and induce severe systemic inflammation, multi-organ failure, and mortality, a syndrome referred to as graft-versus-host disease (GVHD) (5). Although major histocompatibility complex (MHC)-matched transplantation significantly reduces the risk of GVHD, disparity at minor histocompatibility antigens (MiHA) continues to incur risk for GVHD whose target organs include intestine, skin, and liver (5–7). Thus, a matter of great interest is to minimize GVHD, while retaining the anti-tumor response. Particularly strong MiHAs whose expression is limited to hematopoietic cells are attractive targets for accomplishing this goal.

MiHAs arise from the fraction of self-peptides presented conventionally on MHC molecules that happen to be allelically variant (8). Their antigenicity is revealed in transplantation settings because such variant peptides are perceived as foreign to a host's T cells. With the advances in genome wide sequencing and T cell-epitope identification technologies, the number of molecularly identified MiHAs has increased exponentially (9–11). Immunodominant MiHAs have attracted attention as immunotherapeutic targets for hematologic malignancies (12–14). In this review, we describe the molecular features and immunobiology of an unusually immunodominant mouse MiHA, H60, that engender its potent GVL effect.

## H60 and its Immunodominance

Many of mouse MiHAs were identified at the molecular level in the late 1990s and early 2000s (8). Of these, MiHAs for which the specific T cell responses have been functionally evaluated are listed in Table 1 (15–25). Although MiHAs are short peptides processed from various proteins, the molecular functions of the native proteins are in general irrelevant to their ability to generate allo-responses. Prototypic MiHA-specific allo-responses emanate from sequence variation within their MHC-presented peptides. The MiHA H60 differs in two respects. First, the native H60 protein serves as a ligand for the NK cell receptor NKG2D (26, 27). However, this function is unrelated to the role of H60 as a MiHA (H60 family proteins are introduced in Box 1). More importantly, H60 differs in that its allogenicity is based on its presence or absence of the transcripts (*H60*^C^ or *H60*^null^ allele; ^C^ represents allelic variant C, and ^null^ represents alleles with no transcripts) (15). Thus, T cells developed in C57BL/6 (B6; H-2^b^) mice, which have the *H60*^null^ allele and, thereby, do not express H60, become activated when they encounter the completely foreign H-2K^b^-LTFNYRNL peptide (H60p) processed from the protein produced by mouse strains carrying the *H60*^C^ allele, such as BALB and 129.

**Table 1:** Mouse minor histocompatibility antigens.

**Name**	**Distribution**	MHC (H2^**b**^)^*^	**C57BL/6 (allele/or X) sequence**	**BALB.B (allele/or Y) sequence**	**Proportions in B6 anti-BALB.B MLC (30)**	**References**
H60 (*H60a*)	Hematopoietic	K^b^	(b) Null	(c) LTFNYRNL	29.1–36.3%	(15)
H4 (*Emp3*)	Broad	K^b^	(a) SGTVYIHL	(b) SGIVYIHL	6.5–26%	(16)
H28 (*IFi44l*)	Interferon-induced	K^b^		(b) ILENFPRL	6–24.3%	(17)
H7 (*H7*)	Broad	D^b^	(a) KAPDNRETL	(b) KAPDNRDTL	5–8%	(18)
H3a (*Zfp106*)	Broad	D^b^	(a) ASPCNSTVL	(a) ASPCNSTVL		(19)
H13 (*H13*)	Broad	D^b^	(a) SSVVGVWYL	(b) SSVIGVWYL	1–4%	(20)
HY-*Uty*	Broad	D^b^	(X:*Utx*) WMHHTVDLL	(Y) WMHHNMDLI	2–2.5%	(21)
HY-*Dby*	Broad	A^b^	(X:*Dbx*) SSSFSSSRASSSRSG	(Y) NAGFNSNRANSSRSS		(22)

*Underline, amino acid variation between strains*.

* *Superscript b indicates b haplotype of H2*.

Box 1 **The native function of H60.**

In terms of molecular function, the native H60 protein serves as a ligand for the NK cell receptor NKG2D (26, 27). After paralog genes (*H60b* and *H60c*) encoding additional NKG2D ligands were identified (28), the original *H60* was renamed *H60a*. However, *H60b* and *H60c* encode proteins exhibiting amino acid variations at multiple sites including the H60p sequence, LTFNHRTL and LTVKYRTL, respectively, and were found to be transcribed in both the B6 and BALB strains (28). Thus, the MiHA H60 (simplified to H60, hereafter) refers to only the *H60a*-encoded protein that induces the potent T cell allo-response in mouse strains with the *H60a*^*null*^ allele (eg., B6).

In a B6 vs. BALB.B pair, a representative example of MHC (H2^b^)-matched allogeneic donor and recipient mouse strains, MiHA number has been estimated up to 88 (29). However, the immunodominance phenomenon focuses the immune responses to fewer antigens, thus simplifying the complexity of the allo-response. Four MiHAs (H60, H4, H28, and H7) account for great majority of the B6 CD8 T cell responses to allogeneic BALB.B cells (30). But H60 stands out in that it accounts for more than 30% of the B6 anti-BALB.B allo-response (Table 1). H60-specific CD8 T cells expand up to 12% of the CD8 T cells in the blood of B6 mice once immunized with BALB.B splenocytes [this is termed B6 anti-BALB.B host-versus-graft (HVG) response] and compete effectively with CD8 T cells for the allo-MHC (H-2^d^) proteins during the B6 anti-haploidentical CB6F1 HVG response (30, 31). H60 immunogenicity is even more intensified in GVHD. The frequency of the H60-specific CD8 T cells surges up to 25% of CD8 T cells in peripheral blood and target organs of BALB.B GVHD hosts (7). H60-specific CD8 T cells also prevail in other H2^b^-matched GVHD pairs, such as B6 BMT to A.BY, LP/J, and 129 strains. This unusual level of immunodominance endows the value of H60 as a model MiHA to manipulate GVH and GVL responses, with growing evidence favoring the uniqueness of H60 as a GVL target, and is discussed subsequently.

## How and When is H60 Immunodominant?

### Hematopoietic Cell-Restricted Expression

Most known MiHAs exhibit ubiquitous expression patterns. However, H60 is only expressed by hematopoietic lineage cells in mouse strains carrying the *H60a*^C^ allele (15, 27). *H60a* transcripts are detected in lymphoid organs including the thymus and spleen, but not in the kidney, brain, and intestine of BALB/c mice (28, 32). Although one report claimed that *H60a* transcripts were found at appreciable levels in some non-hematopoietic tissues such as cardiac and skeletal muscles and skin (28), its expression in non-hematopoieic parenchymal cells has not been validated in allogeneic solid tissue (skin or heart) transplantation models (as will be described below), and could not be confirmed in our laboratory.

**Figure 1:**
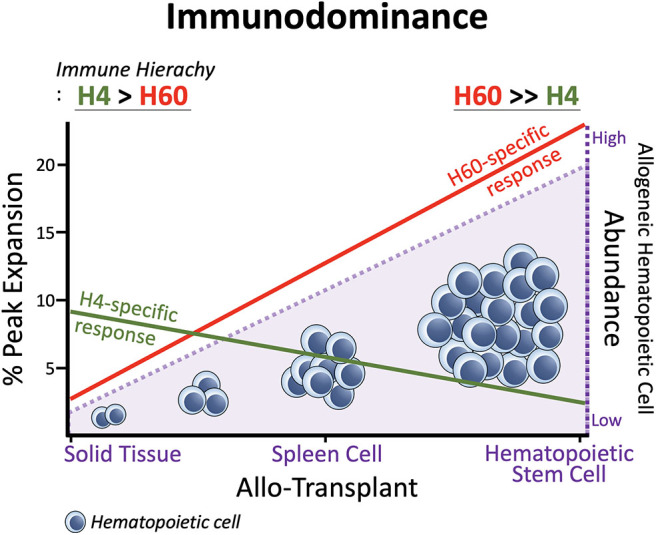
Immunodominance of H60. Expansion of H60-specific CD8 T cells is promoted as the allogeneic hematopoietic cell abundances increase in the order: Solid Tissue, Spleen Cell, and Hematopoietic Stem Cell Transplantations. In contrast, the CD8 T cell response to H4 which represents ubiquitously expressed MiHAs decreases as the allogeneic hematopoietic cells increase. The hematopoieic cell-restricted distribution of H60 facilitates H60 dominance over H4. H60 is dominated by H4 in allogeneic skin transplantation.

In general, H60-specific CD8 T cells undergo robust expansion, attaining peaks of 10–15% of blood CD8 T cells, when B6 mice are immunized with splenocytes from H60 congenic mice (B6.CBy-*H60a*^C^; Con-H60 hereafter) (33–35). However, when tail skin from the Con-H60 strain is transplanted onto B6 mice (Con-H60 → B6), minimal specific T cell expansion is observed (to an average peak of 3%) (36). Similarly, Con-H60 → B6 skin or heart transplantation is associated with minimal skin graft rejection or coronary artery vascular disease (37). This contrasts greatly with the serious complications (almost 100%) found after skin or heart transplantation when the H60-mismatched donor is the H60 transgenic mouse line, C57BL/6 Tg (ACTB-H60a^*^) in which H60 is ubiquitously expressed under the control of the actin-promoter (termed Act-H60 Tg, hereafter) (37, 38). Similarly high rates of complications are observed when the solid tissue transplantations feature mismatch of MiHA H4, a widely expressed MiHA, using the H4 congenic strain (B10.129-H4^b^; Con-H4) as the donor (37). Collectively, these findings indicate that parenchymal cells do not naturally express H60; expression is restricted to hematopoietic cells and the H60-specific response is weak after solid tissue transplantations. In support of this, BALB.B → B6 cardiac engraftment and skin transplantation, neither of which features primary vascularization, H60 is subordinated to H4 (36, 39). However, when the BALB → B6 heart transplantation involves a primary vascularization procedure, so that the BALB.B hematopoietic cells become more exposed to the B6 immune cells, H60 regains its dominance over H4 (H60>H4), as after BALB.B spleen cell immunization (30, 39). Similarly, H60 immunodominance is exaggerated during B6 anti-BALB.B GVH responses (7), when homeostatically proliferating B6 T cells are exposed to a large number of H60-positive host leukocytes. Thus, immunodominance of H60 is flexible and depends on the type of graft. H60 dominance is intensified by the abundance of H60-expressing allogeneic hematopoieic cells, due to its hematopoietic cell-restricted distribution (Figure 1).

### Contribution From High Precursor Frequency in the Naïve T Cell Pool

Various factors affect antigen immunodominance and the immune hierarchy. Affinity for the MHC and the numbers of peptide/MHC complexes are crucial factors influencing immunodominance (40–42). However, the binding affinity of the cognate H60p LTFNYRNL to H-2K^b^ is 3–10-fold lower than that of the Ova_257−264_ SIINFEKL peptide, but is similar to that of the SGIVYIHL H4^b^ CD8 epitope (Kd = 0.8 ± 0.05 nM), and empirical estimates of natural LTFNYRNL/H-2K^b^ complexes (5–15 copies per cell) are not exceptionally high (15, 43). Thus, affinity and ligand density do not readily explain the unusually high immunodominance; H60-specific T cells expand at significant levels (to attain 7–8% of the peripheral peak) even in the presence of allo-MHC responses during the B6 anti-BALB/c (H-2^d^) HVG response, and H60-targeted T cell cytotoxicity is detected at levels similar to that of H-2^d^-targeted cytotoxicity during the haplo-MHC mismatched B6 anti-CB6F1 HVG response (31). H60 immunodominance is reproduced after immunization of B6 mice with the synthetic H60 peptide; naïve protein expression is not involved (31). Rather, precursor cells are significantly frequent in the naïve CD8 T cell pool. The precursor frequencies (ca. 1/24,000–1/11,000 cells) are significantly higher than those of cells recognizing the H13 and HY subdominant MiHAs (such cells are in fact undetectable), and comparable to the level of cells reactive to a viral epitope VSV peptide (RGYVYQGL; ca. 1/49,000–1/18,000 cells) (31, 44). Additionally, the precursor TCR repertoire is diverse as revealed the high shannon entropy (average 5.8) and simpson index (0.99) of rearranged TCRβs sequences (45). Consequently, CD8 T cells with a wide spectrum of TCRs, thus almost all TCRVβs and the various CDR3s of each TCRVβ, are expanded in B6 mice immunized with spleen cells from Con-H60 mice (46). These features may explain why H60 is so immunogenic. However, because boosting the frequency of subdominant H13-reactive T cells via pre-immunization does not attenuate the dominance of H60 (30), not only the frequency and TCR diversity, but also the TCR avidity for H60p/H-2K^b^ of the precursors which are generated through selection processes in the B6 (H60^null^) thymic environment likely play roles in establishing the immunodominance of H60 in the B6 T cell response.

## Does Hematopoietic Cell-Restricted Distribution Affect Thymic Selection of T Cells Specific for H60?

As described above, the immunodominant H60 serves as useful model antigen when studying anti-MiHA allogeneic T cell response in MHC-matched allo-HSCT settings. Anti-H60 donor T cell responses can occur at two different levels. Mature donor T cells contained in the graft inoculum recognize host H60 and induce the GVL effect and GVHD. Also, donor-derived naïve T cells newly developed in the recipient thymus may recognize host H60 to induce the GVL effect and GVHD. Although many studies have focused on acute effects of the former T cells, *de novo* generated naïve T cells also can contribute to both the GVL effect and GVHD. Thus, it is important to understand thymic development of H60-specific naïve T cells. Below, we will describe natural thymic selection of H60-specific T cells and under allo-HSCT settings.

### Incomplete Thymic Negative Selection of T Cells Specific for Self-Hematopoietic Cell-Restricted Antigens

It is well-established that T cells with specificity for ubiquitously expressed self-antigens are deleted in the thymus, preventing T cell-mediated autoimmunity (47). This is true for tissue-restricted antigens (TRAs) that are expressed only in certain peripheral tissues and cells; antigen-specific T cells for TRAs can be negatively selected due to AIRE-mediated promiscuous expression of certain genes (such as endocrine genes including insulin) by medullary thymic epithelial cells (mTECs) (48, 49). However, in recent years, it has become clear that some T cells escape thymic negative selection. In particular, certain TRA-specific T cells, especially those with low avidity TCRs, survive negative selection in the thymus and enter the periphery (50–53). Thymic negative selection against hematopoietic cell-restricted antigens (HRAs), especially natural HRAs such as H60, has not been studied in depth. Thymic dendritic cells (DCs) are known to be responsible for negative selection in the thymus (47, 54, 55). They have great capacity to delete thymocytes with high affinity/avidity TCRs for self-expressed antigens (direct presentation) and also those expressed by mTECs (cross-presentation) (56, 57). The conventional view has thus been that thymic deletion of T cells specific for HRA would be strict because of its thymic DC expression. However, our recent study using the natural antigen H60 as self- and allo-HRA revealed that some HRA-specific T cells survive thymic negative selection (45).

Our initial findings came from experiments using TCR-Tg mice of the B6 (H60^null^) background strain, termed J15 mice, in which all T cells express TCRs originated from a high avidity anti-H60 clone (58). The J15 TCR has high specificity for H60, in that J15 T cells are strictly deleted in the thymus of Act-H60 Tg mice, but not in the thymus of Tg mice where a signal amino acid variant of H60 termed H60H (LTFHYRNL) is expressed under the control of the actin promoter (Act-H60H) (59, 60). Despite the high specificity and avidity, J15 T cells are incompletely deleted in the Con-H60 thymus even though the thymic DCs express H60. CD8 single positive (SP) thymocytes and splenic T cells were generated after crossing J15 and Con-H60 mice, although the numbers were 3–7-fold less and tetramer staining intensity was about 7-fold lower than those of B6 mice. Thus, J15 T cells expressing low avidity TCRs composed of transgenic TCRβ and endogenous TCRαs escaped negative selection in the Con-H60 thymus. Even under physiological conditions, H60-tetramer-binding polyclonal T cells can be detected among CD8 SP thymocytes and splenocytes of Con-H60 mice, albeit with lower tetramer-staining intensities and in numbers 2–2.5-fold lower than those of B6 mice. However, tetramer-binding cells are rarely detected in Act-H60 Tg mice. Thus, the incomplete deletion of T cells in the Con-H60 thymus is attributable to the natural hematopoietic cell-restricted expression pattern of H60. This finding allies with a study showing that limiting TRA expression to DCs results in incomplete deletion of thymic CD4 T cells (52).

### Incomplete Thymic Negative Selection of T Cells Specific for Allo-HRA H60

In an H60-single antigen-mismatched allo-HSCT model, J15 → Con-H60, donor-derived J15 T cells developing *de novo* are also only partially deleted in the thymus of Con-H60 recipients. Because radiation conditioning induces hematopoietic cell death in the recipient, negative selection may have not occurred. Thus, partial deletion in this setting was somewhat surprising. The mediators of such partial negative selection turned out to be radiation-resistant hematopoietic cells of the Con-H60 recipient: J15 T cells were positively selected in the J15 → β*2m*^−/−^Con-H60 BMT, whereas partial deletion was preserved in the β*2m*^−/−^J15 → Con-H60 BMT. Partial deletion of HRA-specific CD8 T cells was also evident when Ova was expressed as an HRA in the recipient of OT-1 BM (OT-1 → [Ova Tg → B6]) (45).

Therefore, deletion of T cells specific for HRAs may not be as strict as was conventionally thought. When limited numbers of DCs serve as the cognate thymic APC, the specific T cells are only partially deleted. Even when all thymic DCs are cognate APCs (as in Con-H60 mice), weak antigen-presentation by each APC (5–15 copies of the antigen-MHC complexes in the case of H60) may prevent strict deletion of the T cells. Thus, the hematopoietically-limited nature of H60 presentation allows low avidity T cells to escape thymic deletion.

## A GVL Effect Mediated BY Post-Thymic CD8 T Cell Escapees Specific for HRA H60

In some animal models of allo-HSCT, hosts with chronic GVHD exhibit mTEC injuries (61–63). In recipients expressing Ova as a TRA, TRA-specific CD4 T cells are generated *de novo* because of mTEC damage occurring during the period of acute GVHD. Post-thymic T cells generated without negative selection trigger autoimmune-like disease in the context of a pro-inflammatory milieu (64). Thus, not just the failure of complete negative selection, but also acute GVHD-associated inflammation increases the likelihood that TRA-specific deletion escapees will generate autoimmune-like chronic GVHD.

**Figure 2:**
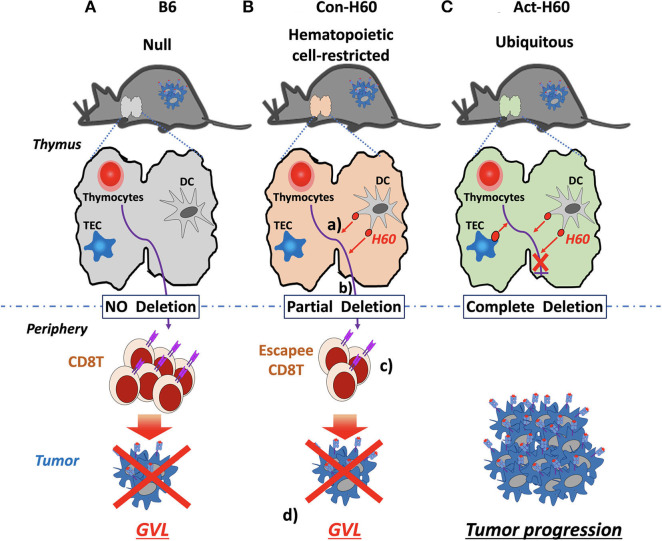
GVL effects by H60 HRA-specific thymic deletion escapees in allo-HSCT. **(A)** In normal B6 recipients (H60^null^), donor-derived T cells (H60^null^ B6) specific for H60 are not deleted in the thymus because H60 expression is lacking. Surviving high-avidity H60-specific T cells mediate a GVL effect against H60-expressing hematologic tumors in the periphery. **(B)** In Con-H60 recipients (that express hematopoietic cell-restricted H60 on the B6 background): (a) donor-derived T cells specific for H60 are partially deleted by H60-expressing host dendritic cells (DCs) in the thymus; (b) low-avidity H60-specific T cells survive this negative selection; and (c) differentiate into effectors in the periphery. (d) These effectors mediate a GVL effect against H60-expressing hematologic tumors. **(C)** In Act-H60 recipients (that express H60 ubiquitously on the B6 background), donor-derived T cells specific for H60 are completely deleted by the H60-expressing thymic DCs and thymic epithelial cells (TEC). In the absence of surviving T cells specific for H60, H60-expressing hematologic tumors cannot be eliminated in an antigen-dependent manner.

In the case of H60, CD8 T cell escapees specific for HRA H60 consist of low-avidity T cells, exhibiting low tetramer-staining intensity. However, their TCR repertoire diversity is comparable to that of the B6 counterparts generated in the absence of negative selection. Escapees generated in B6 → Con-H60 BMT recipients are functional, producing IFN-γ and proliferating in response to H60 peptide-stimulation. More importantly, they have potent anti-leukemia effects. B6 → Con-H60 hosts showed tumor-free survival rates comparable to that of B6 → B6 hosts, because tumor cells were eliminated in an antigen-specific manner (Figures 2A,B). Such GVL effects were not found for B6 → Act-H60-Tg hosts because of the strict deletion of the H60-specific T cells (Figure 2C). Another critical point is that HRA H60-specific CD8 T cell escapees did not cause GVHD-like symptoms in B6 → Con-H60 hosts. However, when the numbers of escapees are non-physiologically high, as in the J15 → Con-H60 BMT, GVHD-like symptoms and mortality were observed in some hosts (<30%). Other findings have included donor leukocyte chimerism and expansion of H60-specific CD8 T cell escapees during the GVL response (45). Such data are clinically relevant as similar phenotypes are observed in leukemic patients showing favorable outcomes after allo-HSCT (12, 65, 66). Thus, hematopoietic cell-restricted MiHA-specific naïve T cells can develop in allo-HSCT hosts and contribute to the GVL effect with minimal GVHD, highlighting the potential utility of hematopoietic MiHA-mismatched HSCT in the clinic.

## GVL Effects Mediated by Donor Mature CD8 T Cells Specific for HRA H60

As we mentioned above, during the early phase of allo-HSCT, donor-derived mature T cells initiate acute GVH, and GVL responses. Donor-derived mature T cells specific for H60 expand greatly soon after transplantation, predominating the B6 anti-BALB.B GVH response (7, 67). However, depletion of T cells specific for H60 (and H4) within the graft prior to transplantation does not alleviate GVHD severity (68), indicating that donor-derived mature T cells specific for HRA H60 are not critical for inducing acute GVHD. It is thus clear that T cells responses raised against multiple MiHA-mismatch, rather than the just two MiHA, contribute to GVHD induction, consistent with the fact that an H4 single mismatch cannot induce acute GVHD (69). However, substantial GVL effects have been observed after transfer of T cells containing memory cells for H60 (70, 71). In MHC (H-2^b^)-matched C3H.SW (H60^null^) → Con-H60 allo-BMT, transfer of CD8 T cells from H60-vaccinated donors, containing 2,600–5,000 H60-tetramer^+^ cells, prolonged the survival of the H60^+^ tumor-bearing hosts with chronic phase or blast crisis chronic myeloid leukemia (CML) (70, 71). The remarkable expansion of the H60-specific T cells (up to 56% of splenic CD8 T cells) and effective tumor killing, compared to the naïve T cell transfer, reflect inclusion in the transplant of central memory cells which can proliferate and differentiate into effector immediately upon antigen-restimulation. In addition to the direct cytotoxic effect, the ability to generate IFN-γ producing effectors immediately and in large numbers allows the memory T cell transfer to exert a powerful GVL effect: IFN-γ sensitizes GVL-resistant blast crisis CML and acute myeloid leukemia to T cell-mediated killing (71). Notably, this memory cell transfer strategy does not generate the GVL effects in hosts where H60 is ubiquitously expressed (70). The memory cells induce only mild hepatic GVHD, unlike the typical aggressive GVHD seen in hosts transferred with naïve T cells. Thus, memory T cells specific for an HRA may serve as tumor-targeting tools mediating a GVL effect. CD4 help is required for appropriate generation and expansion of memory CD8 T cells specific for cellular antigens including H60 (34, 35). Therefore, the development of strategies that include or supplement CD4 help factors will render amplification of memory T cells feasible. Collectively, GVL studies using H60 as a model HRA have validated the use of HRA-mismatched allo-HSCT and HRA-specific memory T cells to maximize GVL effects, while minimizing GVHD, in treatment of hematological malignancies.

## Conclusion

We have reviewed the molecular characteristics of H60, a hematopoieic cell-restricted immunodominant MiHA, and the GVL effects of specific T cells. H60 allelism (*H60a*^null^ vs. *H60a*^C^) and the hematopoietic cell-restricted distribution explain the mechanisms, such as the frequency, diversity, and avidity of reactive T cells, which underlie H60 immunodominance and the GVL effect of H60-specific T cells. Particularly, thymic deletion escapes of T cells with low avidity for HRA H60, and a GVL effect generated by the escapees against H60-positive tumor cells in the periphery have not been evaluated previously. These findings, together with the GVL effect generated by the transfer of memory T cells, emphasize the utility of HRA identification and the use of HRA-mismatched allo-HSCT to treat leukemia and lymphoma.

HRA-mismatched allo-HSCT, and the potential use of human HRAs such as HA-1 (*HMHA1*; VLH/RDDLLEA restricted by HLA-A^*^0201) and HA-2 (*MYO1G*; YIGEVLVSV/M restricted by HLA-A^*^0201) as targets for lysis of leukemic cells have been evaluated in clinic for years (72–75). The HA-1-mismatched allo-HSCT followed by HA-1-negative donor lymphocyte infusions successfully treated a relapse of HA-1^+^ leukemia (12, 76, 77). T cell clones from patients with GVL in the absence of GVHD consistently did not react with non-hematopoietic cells, whereas those from GVHD patients were skewed to broadly expressed MiHAs (78). TCRs from high avidity HA-1-specific clones were used to develop memory T cells targeting leukemia (79). In this respect, identification of HRAs and their specific T cell clones is valuable. HRA expression may not be static, being possibly down-regulated by physiological, or pathological signals including IFNs (80, 81). However, MiHAs afford advantages compared to highly personalized tumor-associated neo-antigens, in that MiHAs shared by a group of people may allow germ-line based treatments. Moreover, the expression of certain HRAs, such as H60 and HA-1 (82–84), can be ectopically induced during carcinogenesis, extending the potential of HRA-based therapies to solid tumor target. Recently, a library of 39 novel MiHAs (restricted by HLA-A^*^02:01 or HLA-B^*^44:03) expressed on hematological cells has been reported (10). Molecular characterizations of these MiHAs and the reactive T cells will aid the utilization of HRAs as GVL targets. Our present review will assist in selection of HRAs for clinical applications.

In summary, mouse studies using H60 as a model HRA have yielded basic knowledge supporting the importance of strong immunogenic HRAs, and donor-derived post-thymic T cells and memory T cells specific for such HRAs for generation of GVL effects. The H60^+^ tumor targeting by post-thymic T cell was revealed using a single antigen-mismatched model. This will be extended to multi-antigen-mismatched allo-HSCT models. Also, future mechanistic studies on GVL mediation by H60-specific T cells will increase our ability to develop strategies that sustain anti-tumor effects while minimizing GVHD or autoimmune-like symptoms.

## Author Contributions

EC wrote the manuscript. KC provided critical insight and edited the manuscript. GN provided supportive experimental data. GN, WK, and MC designed the figures.

## Conflict of Interest

The authors declare that the research was conducted in the absence of any commercial or financial relationships that could be construed as a potential conflict of interest.
